# The Postoperative Component of MAGIC Chemotherapy Is Associated with Improved Prognosis following Surgical Resection in Gastric and Gastrooesophageal Junction Adenocarcinomas

**DOI:** 10.1155/2013/781742

**Published:** 2013-09-17

**Authors:** A. Mirza, S. Pritchard, I. Welch

**Affiliations:** Departments of Gastrointestinal Surgery and Histopathology, The University Hospital of South Manchester, Southmoor Road, Wythenshawe, Manchester M23 9LT, UK

## Abstract

*Aims*. MAGIC chemotherapy has become the standard of treatment for patients undergoing curative resection for gastric and gastrooesophageal junction (GOJ) cancers. The importance of postoperative component of this regimen is uncertain. The aim of this study was to compare survival and cancer recurrence in patients who have received neoadjuvant and adjuvant chemotherapies according to MAGIC protocol with those patients completing only neoadjuvant chemotherapy. *Methods*. 66 patients with gastric and GOJ adenocarcinomas treated with neoadjuvant and adjuvant chemotherapies according to the MAGIC protocol were studied. All patients underwent potentially curative surgical resection. The histological, demographic, and survival data were collected for all patients. *Results*. The median number of neoadjuvant chemotherapy cycles received was 2 (range 1–3). Thirty-one (47%) patients underwent adjuvant chemotherapy with a median of 2 cycles (range 1–3). Patients who have completed both cycles of chemotherapy had significantly improved survival (*P* = 0.04). Patients with involved lymph nodes and positive longitudinal resection margins had increased incidence of recurrence (*P* = 0.02) and poor five-year survival (*P* = 0.03). *Conclusions*. Patients who received both neoadjuvant and adjuvant chemotherapies for gastric and gastro-oesophageal junction tumours have improved outcomes compared to patients who only received neoadjuvant chemotherapy.

## 1. Introduction

The annual worldwide incidence of gastric cancer is approximately one million and is ranked the fourth most common cancer worldwide [1]. There are estimated 7,700 new diagnoses and 5,200 deaths from the disease in the UK annually [2]. In the last two decades, more gastric cancer has been diagnosed in the proximal stomach and around the gastrooesophageal junction (GOJ) [3]. Increased levels of gastrooesophageal reflux disease [4] and obesity [5] are identified as probable causative factors. 

Both gastric and gastrooesophageal junction (GOJ) cancers are associated with poor five-year survival rates [6–8]. Several treatment strategies have been developed to improve outcome. Potentially curative treatments involve surgical resection and both neoadjuvant and adjuvant chemotherapies, sometimes combined with radiotherapy [9]. The aim of neoadjuvant treatment is to decrease tumour bulk, improve rates of surgical tumour clearance, and treat occult micrometastatic tumour. Several trials have used a combination of neoadjuvant and adjuvant chemoradiotherapies to improve outcome with varied success.

The use of adjuvant chemoradiation in gastric and GOJ adenocarcinomas (ACC) has achieved mixed results [10, 11]. However, use of neoadjuvant chemoradiotherapy has been shown to improve outcome in GOJ ACC [12]. In gastric cancer, neoadjuvant and adjuvant chemotherapies have been shown to improve overall survival [13, 14]. 

The landmark MAGIC chemotherapy trial conducted by the MRC (UK) has established guidelines for the administration of perioperative chemotherapy (cisplatin, 5-fluorouracil (5-FU), and epirubicin) in the surgical management of gastric and GOJ ACC [15]. The study recruited patients with gastric, gastrooesophageal junction, and lower third of oesophageal tumours. 45 centres in the UK, Europe, and Asia participated in this randomised control trial (RCT). Between 1994 and 2002, 503 patients were randomised to receive perioperative chemotherapy and surgery (*n* = 250) or surgery alone (*n* = 253). 65% (*n* = 137) of patients started adjuvant chemotherapy but only 42% (*n* = 104) completed all six cycles of perioperative chemotherapy. The results showed overall improved survival of 36% in the chemotherapy groups versus 23% in surgery-only group on an intention to treat basis. The results also showed improved progression-free survival in perioperative chemotherapy group. The study mentioned a problem of lack of clarity and information regarding chemotherapy, whether it was neoadjuvant or adjuvant chemotherapy or a combination of both responsible for improved overall survival and progression-free survival. The study did not publish the survival comparison for patients receiving both neoadjuvant and adjuvant cycles versus patients who only received neoadjuvant chemotherapy. 

Presently, in the UK it is standard practice to offer perioperative chemotherapy for gastric and GOJ ACC for appropriate patients [15]. The aim of this study was to review the outcome for patients who have received MAGIC chemotherapy for gastric and GOJ ACC at our institute. Specifically we aimed to assess survival differences in patients completing perioperative chemotherapy compared with patients who did not complete neoadjuvant chemotherapy.

## 2. Methods

### 2.1. Patient Characteristics

A total of 272 patients underwent surgical resection for gastric (*n* = 115) and GOJ (*n* = 157) ACC between 1996 and 2010 at the University Hospital of South Manchester. 66 of these patients received neoadjuvant chemotherapy for gastric and GOJ ACC according to MAGIC chemotherapy protocol and subsequently underwent surgical resection. Inclusion criteria were histological diagnosis of ACC, locally advanced disease (T1 to T4, N0 to N2, and M(0)), and fit for both surgical resection and perioperative chemotherapy. All patients underwent a standard staging CT scan (chest, abdomen, and pelvis). The positron emission tomography (PET CT scan) was performed for patients with distal oesophageal and GOJ tumours. Endoscopic ultrasonography has been used for local staging since 2006. Staging laparoscopy was performed in all cases. All patients underwent cardiopulmonary exercise testing as part of preoperative assessment for fitness for general anaesthesia. All cases were discussed in a multidisciplinary team. A consensus decision to offer neoadjuvant chemotherapy was made, and patients were counselled accordingly. All patients were treated according to MAGIC protocol [15]. The chemotherapy was administered in three pre- and three postoperative cycles. Each cycle consisted of epirubicin (50 mg/m^2^) by intravenous bolus and cisplatin (60 mg/m^2^) intravenously with hydration on day one and 5-FU (200 mg/m^2^) daily for 21 days by continuous intravenous infusion. A full blood count, serum electrolyte profile, serum creatinine, coagulation, and liver function test monitoring were performed during each cycle. Patients were closely monitored for development of side effects of chemotherapy. In patients with a history of ischaemic heart disease, echocardiogram was performed to assess the left ventricular function. The dose of chemotherapeutic agents was modified in patients with myelodepression, thrombocytopenia, and compromised renal function. A restaging scan was performed following completion of neoadjuvant chemotherapy. In the absence of further disease progression patients underwent total or subtotal gastrectomy and D2 lymphadenectomy depending upon the tumour site. Patients with GOJ tumours underwent total gastrectomy or Ivor-Lewis oesophagectomy as appropriate. Postoperatively patients were managed by a multi-disciplinary team on the high dependency unit and then on a surgical ward. Following recovery from surgery, patients were reassessed for suitability to receive adjuvant chemotherapy. All patients were reviewed in the outpatient clinic, with progress closely monitored.

The demographic details of patients survival status, disease recurrence, and followup were recorded. The postoperative survival was analysed from the date of surgery to last followup or death. The time to recurrence was calculated from the date of surgery to the radiological and clinically proven evidence of disease recurrence.

### 2.2. Histological Data

A data set was developed to collect histological information for patients who underwent surgical resection. This included site of tumour, local stage (T), nodal status (N), metastases (M) according to TNM 5 classification, differentiation, and status of longitudinal resection margins. 

### 2.3. Statistical Analysis

SPPS version 16 (SPSS, Chicago, IL, USA) was used for statistical analyses. The histological characteristics including ypTNM, histological grade, and resection margins status were compared against the survival. The survival curves were obtained employing the method of Kaplan-Meier. Both the univariate and multivariate analyses were performed using the log-rank test. A *P* value of < 0.05 was considered statistically significant.

## 3. Results

Data was collected for the 66 patients who received neoadjuvant chemotherapy according to MAGIC protocol.  Table 1 summarises the characteristics of the study population. The median age was 63 years (range 36 to 76 years). The median number of neoadjuvant and adjuvant cycles completed was 2 (range 1 to 3). 

Thirty-one (47%) patients received both neoadjuvant and adjuvant courses of chemotherapy (Table 2). In 11 (17%) patients, who completed full course of chemotherapy (neoadjuvant and adjuvant) the median postoperative survival was 14 months (95% Confidence interval (CI), 12–28) and time to recurrence was 12 months (95% CI, 12–28). In patients who completed only the neoadjuvant chemotherapy the median adjuvant survival was 8 months (95% CI 11–25) and time to recurrence was 7 months (95% CI, 9–19). In our study 35 (53%) patients did not receive adjuvant chemotherapy. This was because of postoperative complications, patient refusal, or time lapse between surgery and initiation of adjuvant chemotherapy. There was no significant difference in the rate of recurrence between the two groups. Only three (5%) patients showed complete histological response to neoadjuvant treatment as defined by histological analysis of resected specimens (two patients completed full three cycles and one patient received only two cycles). The univariate and multivariate analyses identified completion of both neoadjuvant and adjuvant chemotherapy courses (HR (hazard ratio) 0.26, *P* = 0.008), nodal status (HR 1.20, *P* = 0.014), and longitudinal resection margin status (HR 1.35, *P* = 0.015) as independent markers of prognosis. Table 3 details the chemotherapy-related side effects and grading of symptoms.

**Figure 1:**
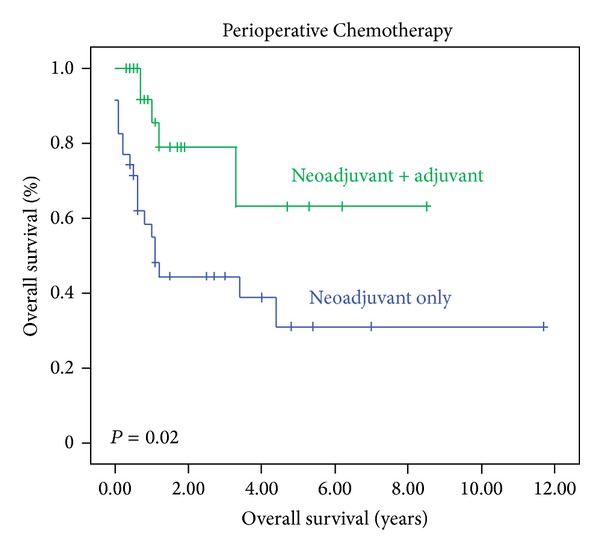
The overall survival comparing patients who completed both neoadjuvant and adjuvant chemotherapy courses versus patient who received only neoadjuvant chemotherapy.

The Kaplan-Meier plot showed significant survival difference between patients completing the neoadjuvant and adjuvant chemotherapies compared with patients receiving only neoadjuvant chemotherapy (*P* = 0.02) (Figure 1). The involvement of the nodes (*P* = 0.004) and longitudinal resection margins (*P* = 0.03) by the tumour were associated with poor outcome.

## 4. Discussion

This is the first study to investigate the survival outcome difference among patients receiving chemotherapy according to MAGIC protocol (Figure 1). Patients who received both neoadjuvant and adjuvant courses had prolonged survival. There was no statistical difference in the rate of incidence of recurrence between the patients completing both neoadjuvant and adjuvant courses of chemotherapy and patients only receiving neoadjuvant chemotherapy (Table 1). However, recurrence occurred sooner in patients who received only neoadjuvant chemotherapy, although only 11 (17%) patients completed all six courses of perioperative chemotherapy. Also in these two groups of patients no significant difference was observed in terms of neoadjuvant staging and medical fitness before the initiation of chemotherapy.

The difference in postoperative survival may signify the oncological importance of completing the full course of perioperative chemotherapy in the absence of prolonged morbidity following surgery. Though the number of patients included in this study was limited to the experience at a single centre. In the future, a study involving multiple centres with a larger cohort can help to explain if the difference in survival is related to one adjuvant cycle or the completion of all three cycles. 

In the management of gastric and GOJ ACC several interventions including neoadjuvant chemotherapy alone, perioperative chemotherapy, and adjuvant chemotherapy alone have been suggested. There is no consensus on the single best treatment option. Specialist centres around the world adopt management strategy which best suits the local practice and guidelines based on the best available evidence.

In 1970s the first Phase I trial of neoadjuvant infusion of chemotherapeutic agents was carried out in Japan [16, 17]. FAMTX trial (5-FU, doxorubicin, and methotrexate) was the first study to randomise patients into neoadjuvant and surgery alone [18]. This trial did not show any clinical benefit but laid the foundations for further research in neoadjuvant settings for management of gastric cancer. The MAGIC trial has widely been recognised as the first landmark study to report the prognostic benefit of perioperative chemotherapy in a large cohort of patients [15]. A second well-reported trial conducted by Fédération Francophone de la Cancérlogie Digestive (FFCD) group showed significant increase in disease-free survival (34% versus 21%) and overall survival (38% versus 24%) over 5 years following perioperative administration of 5-FU and cisplatin in gastric and distal oesophageal ACC [14]. A meta-analysis of 14 neoadjuvant chemotherapy trials in gastric and GOJ ACC concluded improved overall survival (OR = 1.27, 95% CI: 1.26–2.33) and R0 resection rate (OR = 1.51, 95% CI: 1.19–1.91) in patients treated with perioperative chemotherapy.

In Japan following the publication of ACTS-GS trial adjuvant chemotherapy has become the standard of care. The chemotherapy consists of adjuvant administration of cycles of S-1 (orally active fluoropyrimidine). The study showed overall survival rates of 80.1% in the chemotherapy group and 70.1% in the surgery-only group. Furthermore patients who received chemotherapy had less incidence of recurrence [19]. A recently published meta-analysis by GASTRIC group of 17 trials of adjuvant chemotherapy identified improved overall survival (hazard ration (HR) 0.82, 95% CI 0.76–0.90, *P* < 0.001) and disease-free survival (HR 0.82, 95% CI 0.75–0.90, *P* < 0.001) in gastric cancer patients who were administered fluorouracil-based regimen as compared to surgery-only group [20]. On the literature search five randomised control trails (RCT) were identified which have compared one adjuvant chemotherapy regimen against the other [21–25]. Three trials failed to demonstrate an actual difference in overall and disease-free survival [21, 23, 25]. However one trial showed significant improvement in overall and disease-free survival when administering cisplatin, epirubicin, leucovorin and folinic acid versus etoposide, leucovorin, and folinic acid [22]. Similarly another RCT reported improvement in disease-free survival following administration of 5-FU, folinic acid, irinotecan, docetaxel, and cisplatin versus mitomycin-C [24]. In Japan, an ongoing SAMIT study is evaluating the use of paclitaxel and S-1 versus oral tegafur-uracil (UF) for gastric cancer in the adjuvant settings [26]. The final efficacy results are still awaited [27]. The recently published results of CLASSIC trial in patients with stages II and III gastric cancers have identified improved disease-free survival 74% (95% CI, 69–79) in patients administered adjuvant capecitabine and oxaliplatin versus surgery-only group 59% (95% CI, 53–64) [28].

Adjuvant chemoradiotherapy (CRT) has also been employed in the management of gastric cancer. The INT 0116 trial of gastric and GOJ tumours identified improved three-year survival (50%) and disease-free survival (48%) following administration of adjuvant 5-FU, leucovorin, and radiation (45-Gy for 5 weeks) [10]. The group recently published 10-year follow-up results which showed benefit for the CRT group both in terms of overall survival (HR 0.76, *P* = 0.004) and disease-free survival (HR 0.66, *P* < 0.001) [29]. A large RCT CRITICS study is being conducted by a Dutch group. The study aims to evaluate the role of neoadjuvant chemotherapy, surgery and adjuvant CRT versus neoadjuvant chemotherapy, surgery, and adjuvant chemotherapy [30]. A recently concluded RCT of adjuvant chemotherapy versus adjuvant CRT in gastric cancer failed to show any survival difference and clinical benefits between the two groups [31]. Fiorica et al. published a meta-analysis of nine RCT: four trials of neoadjuvant radiotherapy and five of adjuvant CRT [32]. The study reported that reduced mortality in neoadjuvant radiotherapy to surgery alone over 5 years (OR 0.62, 95% CI 0.32–0.64, *P* < 0.00001) was observed when adjuvant CRT was compared to surgery alone. No trials in the literature were identified which have only compared surgery versus adjuvant radiotherapy. 

There is a general consensus that neoadjuvant chemotherapy is well tolerated and tolerance to adjuvant chemotherapy is limited by general morbidity following surgery [33]. Currently STO3 trial conducted by MRC (UK) is employing bevacizumab, a monoclonal antibody targeting vascular endothelial growth factor-A in combination with epirubicin, cisplatin, and capecitabine (ECX) chemotherapy in gastric, GOJ, and lower oesophageal tumours [34].

## 5. Conclusion

Our study showed considerable prognostic benefit achieved following completion of both neoadjuvant and adjuvant chemotherapy courses. Our study included patients who have received chemotherapy and undergone surgical resection. It does not include patients who received neoadjuvant chemotherapy but did not progress for surgery or patients who received chemotherapy and were found to have unresectable disease. Our study is limited by the total number of patients. Our study is limited by the total number of patients and it will be difficult to draw a firm conclusion. However, this study highlights the importance of completing the perioperative chemotherapy. Long-term follow-up results from MAGIC chemotherapy trial are still awaited. This will further lead to a discussion of whether the neoadjuvant or adjuvant chemotherapy is the best option or a combination of chemotherapy actually improves prognosis. 

## Figures and Tables

**Table 1:** Patient characteristics.

Characteristic	No. (%) of patients
Gender	
Male	49 (74%)
Female	17 (26%)
Tumour differentiation	
Well	3 (5%)
Moderate	26 (39%)
Poor	37 (56%)
Tumour site	
Gastric	24 (36%)
GOJ	42 (64%)
T stage	
0	2 (3%)
1	6 (9%)
2	21 (32%)
3	34 (51%)
4	3 (5%)
N stage	
Node negative	19 (29%)
Node positive	47 (71%)
Longitudinal resection margins	
R 0	53 (81%)
R 1	13 (19%)
Tumour recurrence	
Yes	20 (30%)
No	46 (70%)
Neoadjuvant + adjuvant Chemo.	7 (11%)
Neoadjuvant chemo. only	13 (19%)

GOJ: gastro-oesophageal junction; chemo: chemotherapy.

**Table 2:** Number of chemotherapy cycles completed by patients in the perioperative period.

No. of cycles	Neoadjuvant chemotherapy (*n* = 66)	Adjuvant chemotherapy (*n* = 31)
One	12	7
Two	29	13
Three	25	11

**Table 3:** Grading of chemotherapy-related side effects.

Symptoms	Grade (*N*)				
	0	1	2	3	4
Nausea		10	12	2	
Vomiting		3	4	1	
Mucositis		7	1		
Myelosuppression		4	3		
Skin infection		3	1		
Diarrhoea		3	1	1	
Phlebitis		3			
Pancreatitis		1			
Tinnitus		1			
Acute renal failure		1			

*N*: number of patients.
